# Systems modeling and simulation applications for critical care medicine

**DOI:** 10.1186/2110-5820-2-18

**Published:** 2012-06-15

**Authors:** Yue Dong, Nicolas W Chbat, Ashish Gupta, Mirsad Hadzikadic, Ognjen Gajic

**Affiliations:** 1 Multidisciplinary Epidemiology and Translational Research in Intensive Care, Mayo Clinic, Rochester, MN, USA; 2 Mayo Clinic Multidisciplinary Simulation Center, Rochester, MN, USA; 3 Philips Research North America, Briarcliff Manor, NY, USA; 4 School of Business, Minnesota State University, Moorhead, MN, USA; 5 Department of Software and Information Systems, University of North Carolina, Charlotte, USA; 6 Department of Internal Medicine, Division of Pulmonary and Critical Care Medicine, Mayo Clinic, 200 1st St. SW, Rochester, MN, USA

## Abstract

Critical care delivery is a complex, expensive, error prone, medical specialty and remains the focal point of major improvement efforts in healthcare delivery. Various modeling and simulation techniques offer unique opportunities to better understand the interactions between clinical physiology and care delivery. The novel insights gained from the systems perspective can then be used to develop and test new treatment strategies and make critical care delivery more efficient and effective. However, modeling and simulation applications in critical care remain underutilized. This article provides an overview of major computer-based simulation techniques as applied to critical care medicine. We provide three application examples of different simulation techniques, including a) pathophysiological model of acute lung injury, b) process modeling of critical care delivery, and c) an agent-based model to study interaction between pathophysiology and healthcare delivery. Finally, we identify certain challenges to, and opportunities for, future research in the area.

## Review

### Introduction

Modeling and Simulation (M&S) has been commonly used in various scientific domains including ecology, social sciences, economics, and engineering [1]. Modeling has been used by healthcare professionals to investigate disease mechanisms and design novel pharmaceutical agents. In industry sectors, such as manufacturing, aviation, and logistics, M&S techniques have led to major improvements in decision making, efficiency, and quality [2-5]. A real system is modeled to understand its behavior [1]. After building a model from observation or knowledge of a real system, we then test it—a process that is known as simulation. Simulations allow for testing different scenarios, and their results provide explanations for the behavior of the real system and can evaluate various strategies for effective and efficient system operation. The simulation results often indicate the quality of the model itself and may give insight for how to improve its accuracy. A model may be used to highlight areas of system deficiency and predict the impact of proposed interventions without interfering with the normal functioning of a complex system.

Novel medical applications of M&S include studying disease and physiologic processes, predicting and examining human performance, and conducting system evaluations in complex, high-risk healthcare environments [6]. Simulation also has contributed significantly to better training and assessment of clinical and procedural skills; however, this has been extensively reviewed and is not the focus our discussion [7,8]. In this review, we summarize current developments of computer-based M&S applications as they may apply to critical care medicine.

### Rationale for using systems modeling and simulation to improve critical care outcomes

Sir Cyril Chantler stated that “Medicine used to be simple, ineffective and relatively safe. Now it is complex, effective and potentially dangerous” [9]. The intensive care unit (ICU) is an extremely diverse environment with multidisciplinary, multispecialty team members providing care to critically ill patients with complex diseases, using advanced treatment options and technology, under the constraints of physical and electronic infrastructure, equipment, supplies, and processes. All of these elements increase the risk of error and potential threat to patient safety in the ICU [10-13].

Historically, critical care outcomes have been predominantly attributed to the patient’s genetic predisposition, baseline dysfunction, and severity of insult. Data have shed light on the importance of an additional factor: faulty healthcare delivery. Epidemiologic data suggests that delayed or overly aggressive treatments and iatrogenic complications are among the most important drivers of multiorgan failure and poor outcomes during critical illness [14,15].

**Figure 1:**
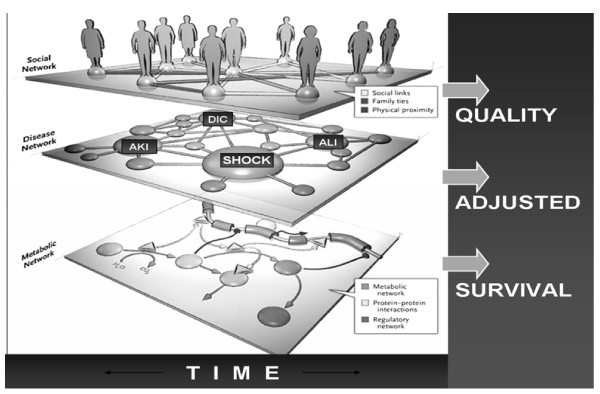
**Network medicine approach, adapted from Barabasi et al. [**[16]**].** AKI, acute kidney injury; ALI, acute lung injury; DIC, disseminated intravascular coagulation.

The ICU is a typical example of a complex adaptive system where collections of interacting components (genome, cells, organ systems, patient, family member, providers, hospitals) react to environments and other agents across different hierarchical levels (Figure1) [16]. Investigating the relationship between specific exposures and outcomes of critical care medicine is challenging due to complex interactions between patient physiology, psychosocial status, and the corresponding healthcare delivery. Different systems’ components interact across varying time scales (seconds to decades) and different spatial scales (from molecular biology to national healthcare policy) within the constraints of physical and cultural environments. The inferences from conventional experimental reductionist and heuristic approaches are limited and even impractical; therefore, novel holistic systems-based approaches are clearly needed. It has been increasingly recognized that the application of simulation methods can offer a novel approach to address the current multifaceted challenges confronting healthcare [5].

Grimm and colleagues provide an outstanding review of modeling approaches for complex- systems analysis [17]. One of the key premises in complex-systems analysis is that knowledge of the elements (agents) that make up a complex system is necessary, but by no means sufficient to understand systems behavior and its impact on (clinical) outcomes. Identifying and deciphering the rules of association and integration within a system are needed if significant breakthroughs in critical care medicine are to occur [18,19]. Researchers collect relevant information about elements at different resolutions, formulate theories about their behavior, implement these theories in a computer model, and, through simulation, observe the emergence of system-level properties related to particular hypotheses. Multiple patterns observed in real systems at different hierarchical levels and scales are used to optimize systematically model complexity and reduce uncertainty.

The availability of quantitative data and the short course from the onset of critical illness to outcome (~60 days) are the clear advantages of implementing M&S methodology to critical care medicine, because it allows for ready validation of hypotheses. Rapid turnaround from the onset of critical illness to outcome and an extremely data-rich environment make the ICU a superb setting in which to explore and develop the capabilities for applying M&S methodologies. One of the first applications of such an approach could be to improve the design and conduct of critical care clinical trials. Currently, nearly all clinical trials in critical care medicine are “doomed to fail” as described in a recent systematic review of randomized trials in critical care by Vincent, due to either naive hypotheses or lack of systems understanding of critical illness and care delivery [20].

Modeling and Simulation can enhance the views of clinical researchers by providing better-suited, more precise hypotheses to be tested in future experiments. In fact, preliminary studies by researchers at the University of Pittsburgh suggest that “in silico” modeling could have prevented universally disappointing results of anticytokine trials in patients with sepsis by pointing out the critical importance of timing of experimental intervention in relation to infectious insult [21]. Similarly, both the timing of interventions tested in current animal models and the lack of appreciation of multiple covariates (multiple hits) in clinical practice preclude the meaningful progress of translational research in acute lung injury (ALI). M&S can provide an invaluable platform for 1) designing clinical experiments, 2) process improvement of care delivery, 3) forecasting and decision support at the patient’s bedside, and 4) informing healthcare policy decisions.

Because a detailed discussion of M&S tools is beyond the objective of this paper, we will instead focus on 1) M&S objective metrics, approaches, and tools, and 2) examples of M&S applications used to study interactions among system components (disease syndromes, patients, providers, and processes) in critical care.

### Common modeling and simulation applications in critical care

**Figure 2:**
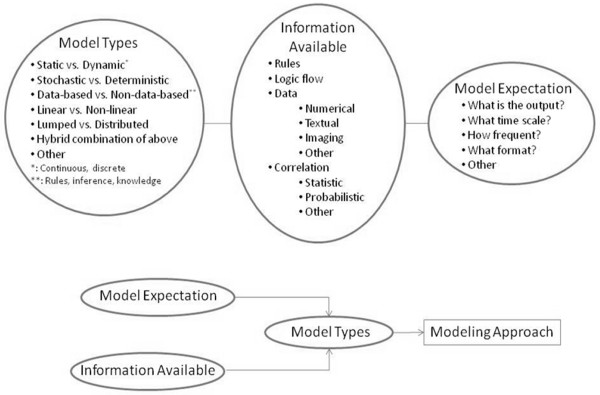
Elements for modeling approach selection.

Thanks to the continual advancement of computer and information technologies, success of advanced engineering methodologies and increased electronic medical record adoption, healthcare sectors are increasingly interested in unleashing the full potential of M&S. The taxonomy of modeling approaches is wide, and the type of modeling approach one chooses to employ depends largely on which component of information about the healthcare delivery system is available (patients, providers, hospitals), its form and fidelity. The upper half of Figure2 displays the three major classes of model types, available information types, as well as model expectation categories. The lower part of Figure2 shows how the expectation from the model to be built, along with which information is available, allows for the selection of the model type. For instance, a certain patient’s disease prediction in time is sought. The available information includes clinical rules, logic flow of disease progression in time, as well as a live stream of numerical ICU data at a certain frequency. Given this set of data, a possible modeling approach would be a hybrid combination of discrete dynamic equations along with an inference engine, working with a real-time parameter estimation scheme. The different model types refer to different mathematical classes of representation. A modeling approach involves a model type along with accompanying mathematical methods in order to render, for instance, a generic mathematical model fine-tuned to an individual patient.

M&S applications can then provide systems- or data-based decision support that would lead to defining a new therapeutic target, predicting patient’s health status or conducting system evaluations of ICU workflow. M&S also offers several distinct gains in learning opportunities that cannot be achieved by traditional approaches [22,23].

#### Monte Carlo simulation & Markov simulation

Monte Carlo simulation (MCS) is a commonly used method for modeling complex systems having recursive processes and events that are impractical and time-consuming to test in the physical world. It generates numerous output scenarios by repeatedly picking random samples from an uncertain variable based on probability distribution. For instance, this mathematical tool has been used in large-cohort studies to assess whether regionalized intensive care could improve the outcome of patients who require mechanical ventilation [24]. Simulation of time-dependent probabilities of bacterial spread offered new means for testing various intervention strategies (antibiotics and infection control) in critical care practices [25]. MCS has been used with optimization techniques to enhance clinical decision making by maximizing antibiotics dosing [26,27]. Markov simulation is another random-process modeling tool that often is used for economic evaluations when comparing different outcomes of complex medical interventions [28]. It has been used to investigate ICU clinical decision making by revealing evidence for sex-based risk difference in ICU patients [29]. Critical care applications of these tools have been recently reviewed by Kreke et al. [30].

#### Discrete event simulation

Discrete event simulation (DES) is among the most commonly applied stochastic analysis tools. DES provides the user with a “test-bed” to perform experiments via computer modeling and test the likely effectiveness of different solutions before their implementation. Different “what-if” scenarios can then be performed in a “prototype” testing environment. A key aspect of DES is the system-state description, which includes values for variables of the system components: probability distribution of entity arrival, event duration, event status, and resources needed. It provides a novel process evaluation mechanism grounded in scientific principles for conducting healthcare system analysis, simulation-based drills, and workflow redesign. DES has been used in the healthcare industry to support clinical decision making, facility planning (to predict bed occupancy), resource allocation (staffing), treatment evaluation, emergency room organizational redesign, and ICU information system usability tests [31-36]. Such applications demonstrate that M&S could be a key enabler for realizing transformation in complex healthcare delivery systems.

#### System dynamics

System dynamics (SD) is a computer-based approach for performing policy evaluation and decision making in systems, such as ecological settings and business processes that exhibit dynamic complexity [37]. The characteristics and behaviors of systems that exhibit dynamic complexity are typically governed by a large number of factors. For example, dynamic systems may exhibit different change in their structure or behavior over varying time scales. Similarly, degree of connectedness, nonlinearity, degree of adaptiveness, self-organizing behavior, complexity of feedback structure, and systems dependence on historical states are just some of the factors that generate dynamic complexity within a system [38]. SD is centered on feedback control theory that uses causal a feedback loop mechanism (that describes the relationship between stocks and flows) to describe either mathematically or graphically endogenous changes that occur within a system. The combination of both qualitative and quantitative approaches makes SD useful at the strategic level [39]. The capacity to describe systems that change over time and generate feedback is central to advanced engineering methodologies and is applied ubiquitously across industrial fields. It also has been used extensively to perform macro-level policy analysis, such as in the area of public health and health policy [40]. The application of SD in critical care remains underutilized and should be further investigated.

#### Agent-based simulation

Agent-based simulation (ABS) is a stochastic simulation in which the local interactions of collections of individual decision-making entities (agents), following simple rules, result in complicated and often nonobvious patterns at the aggregate level [41]. The technique has been used extensively for simulating complex adaptive systems (CAS) in ecology and social sciences [17]. An ABS, when coupled with a robust data resource that provides detailed and reliable essential data elements (patient, provider, and setting characteristics from onset of acute illness through outcome), makes it possible to model critical diseases in a bottom-up manner. Instead of building a single regression model to predict the entire range of behaviors in one simplified relationship, ABS seeks to capture emergent phenomena from individual agents. It represents low-level individual behaviors from different scales (e.g., organ system, hospital) that together result in the observed high-level patterns—with multiple dimensions [41]. Simply put, traditional modeling describes the forest as an indivisible entity, whereas CAS modeling describes the trees and allows the concept of a forest to emerge as a consequence.

### Modeling pathophysiologic processes

The complexity of the disease, insufficient understanding of the evolution and progress of risk factors, and numerous other modifiers severely limit the capabilities of clinicians and researchers to predict disease development and progression and to define appropriate therapeutic targets. In addition to clinical knowledge-based rules, variables, such as genome, disease burden, physiological parameters and conditions, and clinical research evidence, provide conditional probability distributions for modeling logic outputs from organ status to clinical syndromes and outcomes. Due to the complexity of human biology, a bottom-up integrative approach combining data from different levels (genes, molecules, cells, organs, body, etc.) is of limited help in understanding disease pathophysiology [42]. Such approaches are based on mathematical models of disease development. These models can serve many purposes, such as understanding disease mechanisms, disease detection, disease prediction, and in silico testing of “what-if” scenarios and even clinical trials. As such, these models also can be used in clinical decision-support systems.

**Figure 3:**
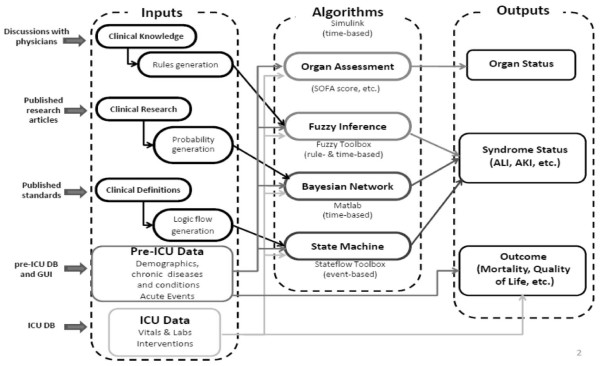
Acute lung injury detection model architecture.

Archimedes (Archimedes, Inc., San Francisco, CA), for example, is a mathematical model that represents the anatomy, physiology, and pathology pertinent to diabetes and its complications [43]. This model has been extensively validated and has already found multiple applications in healthcare policy and research [43,44]. Recently, chronic obstructive pulmonary disease (COPD) models have been developed to offer multilevel approaches to investigate COPD complexity [45]. A new model [46] that was recently developed for ALI detection uses three information sources: electronic medical record (EMR) data, published epidemiologic studies, and mechanistic understanding of domain experts (Figure3). Three modeling techniques were combined: rule-based fuzzy inference, state flow diagrams, and data-based correlation using Bayesian Networks. Clinical ALI development knowledge from ICU physicians were articulated verbally and subsequently written mathematically in terms of linguistic variables and rules. This method leads to a crisp value ALI detection score.

Published research articles from clinical trials reporting odds ratios and confidence intervals were subsequently translated into probability distributions and conditional probability tables feeding a Bayesian Network. This method generates probabilistic a priori knowledge about forward-computing the probability of an event occurring. Finally, pathophysiological understandings of disease mechanisms were translated into event-based conditional rules. A Finite-State Machine (FSM) was then used, because it represents transition from one state to another based on a condition or an event trigger. Different states become active as FSM matures with more data records provided as inputs over time. The last state in the FSM logical flow path is indicative of respiratory failure and ultimately of ALI onset. This type of approach is not data-based, because no data mining or statistical correlations were employed. ICU and pre-ICU data was fed into the developed model to generate ALI detection scores [46]. Other studies also demonstrate the potential of using this approach to address acute inflammatory response and sepsis progression in critical care [47-49].

### Modeling care process for better healthcare delivery

**Figure 4:**
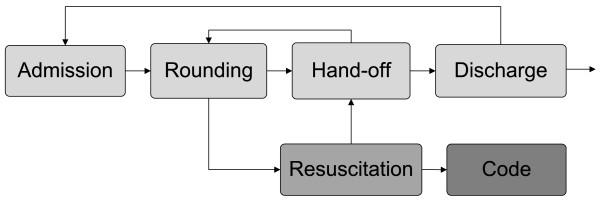
The schematic representation of care process in ICU.

ICU system operations are centered on critically ill patients admitted to units from the emergency room or floor. The ICU interacts with various parts of the hospital system, such as laboratory, transfusion, surgery, interventional and diagnostic radiology services, etc. A lack of clear understanding of critical issues such as workflow variation, resource constraints, communication, etc., precludes us from developing a meaningful understanding of latent factors that cause the sub-optimal performance of an ICU. The concept of systems understanding of care delivery was first introduced by Donabedian, but has been more recently championed by numerous advocates including patient groups, providers and business experts [50-53]. Using multifaceted systems engineering approaches, key ICU processes (admission, resuscitation, rounding, handoff, etc) can be modeled to analyze and modify healthcare delivery in an ICU setting. (Figure4)

**Figure 5:**
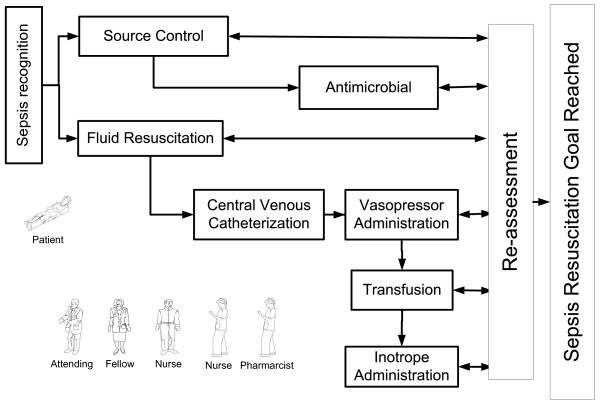
Model of sepsis resuscitation process in ICU.

The process model can be used for several purposes, such as describing the flow of patients admitted to an ICU or predicting the future state of a patient conditional upon the complex interactions of several activities and decisions made in the prior state. Each phase requires an elaborate and in-depth multiscale study of key ICU processes. For example, using a process-modeling approach, a prototype of a DES model of septic shock resuscitation has been developed recently (Figure5) [54]. This model is based on data collected through field observations, electronic medical records, and healthcare provider estimations. Options for improvements in system performance and workflow redesign were then implemented and tested by using a computer simulation model specific to sepsis resuscitation. Several key interventions could be investigated without patient harm: reduction of central line procedure time, modification of laboratory sample draws, etc. The model aims to refine, verify, and validate a comprehensive resuscitation process. This approach also is applicable to other critical care emergencies, including massive bleeding (hemorrhagic shock) or increased intracranial pressure.

### Agent Based Simulation to study the interaction between pathophysiology and healthcare delivery (“nature” and “nurture”)

**Figure 6:**
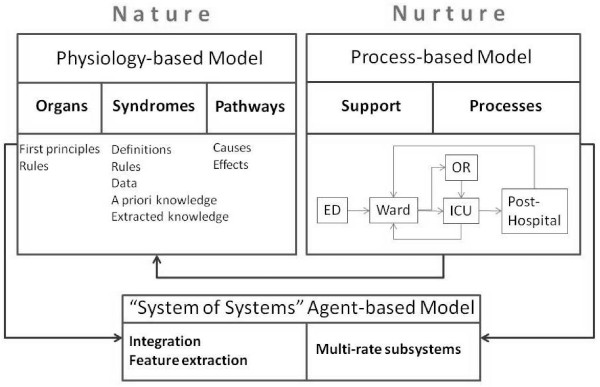
Schematic representation of multiscale modeling approach to simulate acute lung injury and its consequences.

Because of the robust data set from the EMR and various other information sources existing in the modern critical care environment, it is possible to conduct simulations that will produce verifiable streams of data. At the same time, those simulation exercises also can yield additional variables and insights that are not explicitly represented in the data streams. For example, one may detect patterns of disease progression and health thresholds (at the individual patient level), as well as potentially unforeseen consequences of decisions made at the clinical care level in the ICU. Other relationships and system component attributes are then hypothesized, realizing a wider range of possible influences and feedback mechanisms that could account for nonlinear dynamics (for example, a threshold for development of MODS [multiple organ dysfunction syndrome]). “Nature” refer to pathogenesis of acute lung injury. “Nurture” refers to care processes that may be as important as the individual’s biology in development of ALI and MODS (Figure6).

Within an individual patient, for example, there are multiple organ systems that follow physiological rules governing their interactions that aggregate up to the health of that individual. On another level, each patient also engages in a clinical setting consisting of providers and patients that interact as part of treatment, producing a second layer of aggregate (population health and outcome) results. The outcomes of a simulation at one level can both constrain and direct simulations at other levels.

Still more real-time data elements can be added to the model as (hypothetical) aggravating or mitigating effects toward specific organ function, improved process efficiency, or overall patient-centered outcomes. During the course of many simulation runs, the attributes and component relationships that best fit the patient data begin to emerge, allowing for closer approximations of which factors are relevant to patient-centered outcomes and revealing their degree of importance. The inconsequential factors can be eliminated as unnecessary complications to the model. However, by using the complex adaptive systems architecture to simulate these relationships, it is not necessary to be able to predict precisely what these casual connections are *a priori*. By allowing the system to hypothesize the various directions and degrees of causal factors, the model becomes self-calibrating across the time-series data as new patient populations enter the stream, with some segment of that data set aside for testing to guard against overfitting of the data. Once a rigorous general model has been built and tested, then patient-specific data can be input in real-time, which will, ideally, allow the simulation to identify likely outcomes and flag potentially adverse consequences before they occur. For example, to further investigate the host response and complex interactions among cells, cellular networks and organs—as well as to develop treatment options for multifactorial critical illnesses, various M&S applications (including a hybrid approach) have been explored [21,55-57]. ABS methods also have been used for in silico clinical trials to study systemic inflammatory response syndrome/multiple organ failure/acute respiratory distress syndrome using a system-biology approach [58-60].

### Challenges and future recommendations

Several barriers have been identified that have prevented widespread use and adoption of modeling and simulation in healthcare [5,23,61,62]. For example, there are significant disconnects between modeling professionals and healthcare providers, preventing the implementation of proposed models. Furthermore, complexity of both illness and corresponding medical practice, provider-centered rather than patient-centered care, system fragmentation, lack of resources, and limited accuracy and availability of key data elements all hinder rapid application of M&S techniques in critical care and other areas of acute medicine. In addition to multiple layers pertinent to critical illness (from cell metabolism and systems biology to clinical physiology), researchers and policy makers need to consider both “sharp end” (provider education, etc.) and “blunt end” (organizational optimization, etc.) layers of the healthcare delivery system [63,64]. The adoption of the EMR has made patient data (labs, vital signs, notes, etc.) more readily available and will no doubt enhance future modeling approaches (with the addition of large data sets for data mining and predictive modeling) [65]. Further EMR improvements will result in even more meaningful data from various system components of care delivery, such as ICU operational data, provider performance and related process data, as well as more precise account of meaningful patient outcomes (functional status, quality of life, family satisfaction). These are typically stored across several system components but can be collected and analyzed for optimizing disease management [66].

## Conclusions

The National Academy of Engineering and the Institute of Medicine of the National Academies directed attention to the issue of systems engineering, modeling, and simulation application in medicine with their joint report in 2005: “Building a Better Delivery System: A New Engineering/Health Care Partnership” [67]. Recently, the American Medical Association (AMA) and the Institute for Electronic and Electrical Engineers (IEEE) Engineering in Medicine and Biology Society have joined hands for a Second Annual AMA-IEEE Medical Technology Conference in 2011. The unique collaboration between experts in critical care medicine, engineering, research, education, and medical informatics promises to provide clinically relevant systematic approaches and comprehensive solutions to many of the challenging problems in clinical medicine [68]. The M&S-based systematic analysis of both clinical pathophysiology and healthcare delivery will allow for testing of specific interventions (from virtual clinical trials to quality improvement) before clinical deployment, effectively forecasting patient health in the ICU and eliminating potential patient harm. This capacity will offer possibilities to better select high-impact and low-cost interventions for the consideration of future critical care research and quality improvement.

## Misc

This work was supported by the Mayo Foundation for Medical Education and Research, and National Library of Medicine LM10468Z-01

## Competing interests

The authors declare that they have no competing interests.

## Authors’ contributions

YD and OG drafted the manuscript. NWC, AG, and MH added and modified the content. All authors read and approved the final manuscript.
